# Impact of Amyloid Polymorphism on Prion-Chaperone Interactions in Yeast

**DOI:** 10.3390/v11040349

**Published:** 2019-04-16

**Authors:** Andrea N. Killian, Sarah C. Miller, Justin K. Hines

**Affiliations:** Department of Chemistry, Lafayette College, Easton, PA 18042, USA; killiana@lafayette.edu (A.N.K.); millersc@lafayette.edu (S.C.M.)

**Keywords:** Ssa, Ssb, Ydj1, Apj1, Swa2, Sis1, J-protein, Hdj1

## Abstract

Yeast prions are protein-based genetic elements found in the baker’s yeast *Saccharomyces cerevisiae*, most of which are amyloid aggregates that propagate by fragmentation and spreading of small, self-templating pieces called propagons. Fragmentation is carried out by molecular chaperones, specifically Hsp104, Hsp70, and Hsp40. Like other amyloid-forming proteins, amyloid-based yeast prions exhibit structural polymorphisms, termed “strains” in mammalian systems and “variants” in yeast, which demonstrate diverse phenotypes and chaperone requirements for propagation. Here, the known differential interactions between chaperone proteins and yeast prion variants are reviewed, specifically those of the yeast prions [*PSI*^+^], [*RNQ*^+^]/[*PIN*^+^], and [*URE*3]. For these prions, differences in variant-chaperone interactions (where known) with Hsp104, Hsp70s, Hsp40s, Sse1, and Hsp90 are summarized, as well as some interactions with chaperones of other species expressed in yeast. As amyloid structural differences greatly impact chaperone interactions, understanding and accounting for these variations may be crucial to the study of chaperones and both prion and non-prion amyloids.

## 1. Introduction

**Figure 1:**
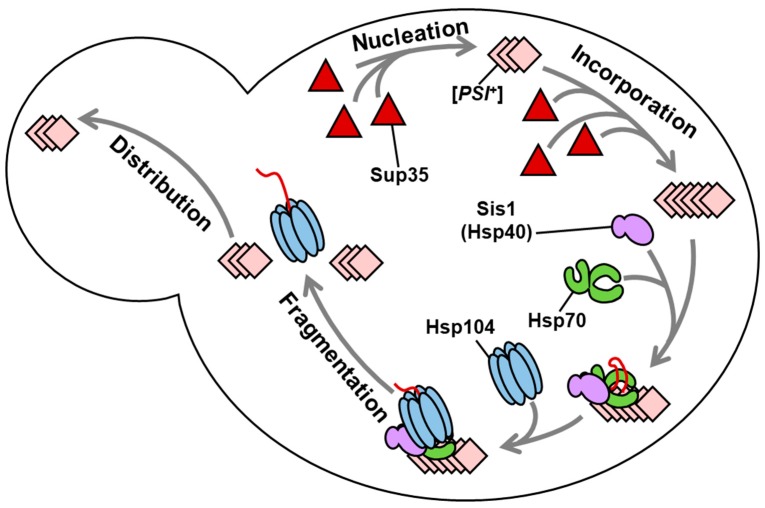
Current model of chaperone-dependent prion propagation *in vivo*. Four interdependent processes are necessary for prion formation and propagation in yeast. Prions arise from a rare event in which protein monomers (represented as triangles in the case of the prion-forming protein Sup35 above) misfold and form a thermodynamically-stable, ordered aggregate (“nucleation”). Aggregates increase in size by recruiting more soluble monomer (“incorporation”). To be transmissible, however, the fibril must also be a target for the chaperone machinery [2]. Hsp40-class chaperones, particularly Sis1, and the Hsp70 Ssa, are thought to functionally recruit the disaggregase Hsp104, responsible for the physical fragmentation of amyloid fibrils to create new propagons (“fragmentation”) that may be inherited by daughter cells (“distribution”).

Yeast prions are protein-based, heritable elements of the yeast *Saccharomyces cerevisiae.* Most yeast prions are amyloid aggregates of functional proteins that become fixed in cell populations by cytosolic spreading of small, self-templating pieces, called propagons, during mitosis [1]. New propagons are created via a biochemical aggregate-fragmentation mechanism involving at least a trio of molecular chaperone proteins: the Hsp40 Sis1, the cytosolic Hsp70 Ssa, and, most importantly, the disaggregase Hsp104 [2]. Current models assert that amyloid aggregates are remodeled and ultimately severed by Hsp104 following the upstream action of Ssa and Sis1 (Figure 1), which facilitate the recruitment and engagement of Hsp104 [3,4,5,6].

A fundamental aspect of amyloid-based prions is the formation of distinct amyloid structures (structural polymorphisms), called “strains”, in mammalian systems [2,7]. Prion strains can be numerous and can exhibit distinct disease pathologies and species transmission barriers in mammals [7]; PrP^Sc^, for example, has been estimated to form over 30 distinct prion strains [8]. Moreover, amyloid polymorphisms are widespread among other amyloid-forming proteins, including amyloid-beta, α-synuclein, transthyretin, and tau, among others [9]. In yeast, the same phenomenon is termed “prion variant” to avoid conflation with the notion of the microbial “strain” of interest. In yeast, prion variants influence the stability of the prion in mitosis, the size distribution of aggregates, the intensity of prion-associated phenotypes, and, as this brief review will discuss, the interactions with various components of the cellular chaperone machinery [10,11,12,13]. We will not attempt to review all known chaperone–prion interactions in the literature, but rather we will focus on highlighting what is known about the differential interactions that chaperones have with distinct variants of the same prion, specifically when it can be reasonably assumed that these observed differences are attributable to prion variation. We omit instances where differential effects have been observed in different lab strains with distinct genetic histories, when the effect of yeast strain genetic polymorphisms may play a role. Therefore, our review is intentionally limited to instances where multiple variants of a prion have been studied simultaneously in the same yeast strains and under presumably identical conditions.

## 2. Prion Variants in Yeast

[*PSI*^+^] is the prion form of the translation termination factor Sup35. Due largely to its distinctive color phenotype, high stability in mitotic cell populations, and long history of study, [*PSI*^+^] has become the most studied yeast prion and arguably the archetype against which others are compared [1]. [*PSI*^+^] variants are typically classified as “strong” or “weak” based on the strength of the loss-of-function phenotype of the prion protein, which is the result of different ratios of soluble to aggregated protein [10]. For many [*PSI*^+^] variants, phenotypic strength generally correlates directly with the stability of the prion in the cell population during mitotic growth. Furthermore, in the few cases where amyloid core size is known, so-called “strong” and “weak” variants are associated with smaller and larger amyloid core sizes, respectively [14], with a recent estimate of twice as many residues involved in the amyloid core of weak variants than with strong variants of [*PSI*^+^] [15]. As expected based on amyloid core size, some strong variants are more physically fragile and more easily fragmented than some weak variants by Hsp104 [16]. Thus, the less stable fibers of these strong variants fragment into more numerous and smaller prion propagons that more efficiently sequester monomeric Sup35, resulting in a stronger phenotype. Likewise, among some strains of PrP^Sc^, smaller aggregates have been found to be more infectious [17], and fiber stability is inversely correlated to incubation periods, indicating that more fragile aggregates may spread more quickly [18,19]. Although often identified only as “strong” or “weak” in many reports in the literature, [*PSI*^+^] variants with these monikers are not necessarily the same, as multiple weak and strong variants have been identified, occasionally behaving differently with respect to chaperone interactions in a few instances noted herein. 

In contrast, for variants of the prion [*RNQ*^+^] (also called [*PIN*^+^]), correlations between phenotypic strength and fiber stability are far less clear. [*RNQ*^+^] variants have been traditionally classified by either the extent to which they induce the formation of [*PSI*^+^] (Pin is an acronym for Psi inducibility) [20,21], referred to as “high”, “medium”, *etc.* [22], or by the aggregate pattern observed when aggregates are tagged with fluorescent probes (typically GFP), referred to as “single dot”, “multiple dot”, etc. [23]. However, Stein and True recently demonstrated that these characteristics are not necessarily associated with fiber thermal stability or fiber fragmentation in vivo [24]. Indeed, [*RNQ*^+^] variants appear to be numerous and diverse, with one estimate being as high as 40 distinct variants [25]. 

Many variants of [*URE3*] have also been described in the literature [26,27,28]. Like [*PSI*^+^], these variants differ in mitotic stability, infectivity, the ability to cross species barriers, and in the intensity of the loss-of-function phenotype of the prion-forming protein, Ure2. Finally, variants of both [*URE3*] and [*PSI*^+^] differ dramatically in the degree of toxicity toward the cell [29], with the most toxic forms eliminated from cell populations. This creates a bias in the variants that are examined in laboratory settings, obfuscating the full diversity of amyloid variation from being explored. Relative to [*PSI*^+^] and [*RNQ*^+^], very little is known about the effects of [*URE3*] prion variation on chaperone interactions. To the best of our knowledge, nothing is known about these effects for any other yeast prions.

## 3. Requirement for Hsp104

Hsp104 is the sole Hsp100-class disaggregase in *S. cerevisiae.* The structure of Hsp104 has recently been solved at near-atomic resolution using cryo-electron microscopy [30]. Hsp104 forms a hexameric ring structure with a central cavity comprised of six AAA+ (ATPases Associated with diverse cellular Activities) protomers, each of which contains an N-terminal domain, two ATPase domains (NBD1 and NBD2) connected by a “middle” (M) domain, and a C-terminal domain [30,31]. As expected of members of the AAA+ family, Hsp104 is only competent as a disaggregase when fully assembled as a hexamer [32]. The M-domain of Hsp104 is dispensable for substrate translocation and ATPase activity but is required for protein disaggregation [33]. Hsp104-Hsp70 interactions have been demonstrated to be required for efficient protein disaggregation, and this interaction is mediated by the M-domain of Hsp104 [33].

Hsp104 is required for the propagation of all known amyloid-associated yeast prions; deletion, chemical inhibition, mutation, and depletion of Hsp104 all result in prion loss [1,34,35,36,37]. Hsp104 is potently inhibited by guanidine ions [38]. That weak [*PSI*^+^] variants are more sensitive to treatment with GdnHCl has been known for more than two decades [10], indicating that weak variants are more sensitive to Hsp104 activity than strong variants. This finding has been confirmed many times [16,36,39,40]. Unsurprisingly, [*URE3*] variants also differ in their sensitivity to GdnHCl treatment [26]. Perhaps the most extreme example of the importance of prion variant structure to Hsp104 requirement is the work of Chernoff and coworkers who reported the isolation of a [*PSI*^+^] variant upon overexpression of Sup35 that was maintainable only at high levels of Hsp104 activity that would normally cure other variants [41].

Mutation of Hsp104 has also revealed variant-specific effects. The True group identified two random point mutations in *HSP104* that disrupted propagation of weak but not strong [*PSI*^+^] variants [42]. Subsequently, they investigated a host of Hsp104 M-domain mutants and observed a general trend with the set of mutants tested and the ability to propagate strong and weak variants [43]. All mutants that supported weak [*PSI*^+^] supported strong [*PSI*^+^] as well, but the converse was not true, indicating that some mutants decreased Hsp104 function to a level that distinguished between strong and weak variants, and only allowed the former to propagate [43]. This indicates the importance of the function of Hsp104’s M-domain, and more specifically, that weak variants of [*PSI*^+^] are more dependent on M-domain function than strong variants [43].

## 4. Hsp70s

Hsp70 molecular chaperones are ubiquitous and involved in a wide variety of protein regulation activities, including protein folding and transport across membranes [44,45]. Hsp70s bind promiscuously to exposed hydrophobic segments on proteins in an ATP-regulated binding cycle. While the Hsp70 binding regions are largely nonspecific, cochaperone J-proteins target Hsp70 action by delivering client proteins or directing Hsp70 to a location (recently reviewed in Kampinga et al. [46]). While there are at least 14 different Hsp70s in yeast, two main subclasses occupy the cytosol: Ssa and Ssb [47]. Both are intricately involved in yeast prion biology, with Ssa in particular being implicated in prion fragmentation [6,48,49]. Differential effects on prion variants have been documented for both subclasses. 

### 4.1. Ssa1-4 (Free Cytosolic Hsp70s)

The Ssa class has four cytosolic proteins, with Ssa1 and Ssa2 expressed constitutively and Ssa3 and Ssa4 induced by heat shock [50]. All four are involved in myriad activities within the cell [44], including prion fragmentation [6]. Overexpression of Ssa1 [51,52,53], or Ssa2 [53], cures weak but not strong variants of [*PSI*^+^] with [52], or without [51,53], Sup35 co-overexpression. Perhaps unsurprisingly then, in another investigation, a weak [*PSI*^+^] variant was slightly more sensitive to the overexpression of a dominant, [*PSI*^+^]-detrimental mutant called *SSA1-21* than a strong variant [39]. Interestingly, the anti-prion effects of *SSA1-21* are dependent on the activity of Hsp90 cochaperones, as are the anti-prion actions of Hsp104 discussed in later sections [54].

The effects of Hsp70 gene deletions on [*PSI*^+^] variants, however, appear to depend on either the specific prion variants examined, or the yeast strain background used. For example, in one investigation in the 74-D694 yeast genetic background, deletion of *SSA2* destabilized a weak variant identified as “OT55” but not a strong [*PSI*^+^] variant called “OT56” [55]. In contrast, a more recent investigation found that in the yeast genetic background 5V-H19, weaker [*PSI*^+^] variants [*PSI*^+^]^VK^ and [*PSI*^+^]^VL^ were lost if *SSA1* was deleted, but the strong variant [*PSI*^+^]^VH^ could propagate in a ∆*ssa1* background. *SSA2*, on the other hand, was not required for [*PSI*^+^]^VK^ and [*PSI*^+^]^VL^ propagation [56]. Interestingly, deletion of *SSA1* also altered the dominance of some variants over others [56].

### 4.2. Ssb1/2 (Ribosome-Associated Hsp70s)

Ssb1 and Ssb2 are non-essential, non-heat inducible, ribosome-associated Hsp70s that are highly similar and functionally redundant. They aid in the proper folding of proteins and are associated with the ribosome with the help of the non-canonical Hsp70 Ssz1 and the cochaperone Hsp40 Zuo1, together forming the ribosome-associated chaperone complex, or RAC [57,58]. Ssb overexpression destabilizes some [*PSI*^+^] variants and promotes Hsp104-mediated [*PSI*^+^] elimination [59,60,61]. Deletion of Ssb, or other protein members of RAC, results in increased [*PSI*^+^] formation, indicating that RAC serves to prevent nascent Sup35 misfolding into the prion form [62,63]. Kushnirov et al. found that overproduction of Ssb1 cured a weak [*PSI*^+^] variant quite efficiently, but not strong variants [59]. A pleiotropic effect may be causal as these authors did find that Ssb1 overproduction reduced Hsp104 levels. Arguably, the decrease in Hsp104 expression alone could explain the loss of weak but not strong [*PSI*^+^] variants. Zuo1 and Ssz1 also have effects on [*PSI^+^*] propagation. Deletion of either increases spontaneous loss of weaker variants of [*PSI^+^*]. An increase in observable prion aggregate size is seen in these deletion strains [62]. 

## 5. Hsp40s (J-Domain Proteins or J-Proteins)

Hsp40s, known also as J-proteins and more recently as J-domain proteins [46], stimulate Hsp70 ATPase activity by virtue of a conserved domain, called a J-domain by orthology to the canonical domain of the *Escherichia coli* protein DnaJ. J-domain stimulation of Hsp70 ATP-turnover enhances client-peptide binding. However, most J-proteins also bind and deliver polypeptides directly to Hsp70s, acting as specificity factors that diversify Hsp70 function [46]. 

### 5.1. Sis1

Four yeast prions, and probably many others, rely on the essential J-protein Sis1 for stable propagation in cell populations [4,39,64]. Despite this apparent universality, requirements for Sis1 activity vary dramatically among different prions and prion variants. 

#### 5.1.1. Sis1 Deletions, Truncations, and Repression

Like most J-proteins, Sis1 has an N-terminal J-domain followed by an unstructured section rich in glycine [44]. This section has been conceptually divided into two regions referred to as the glycine/phenylalanine-rich (G/F) and glycine/methionine-rich (G/M) regions. C-terminal to the glycine regions are two putative peptide-binding domains and a dimerization domain. Multiple studies have now examined the impact of Sis1 domains on distinct variants of [*PSI*^+^], finding that weak [*PSI*^+^] variants are highly sensitive to certain Sis1 truncations or internal deletions, whereas strong [*PSI*^+^] variants are generally unaffected [40,65,66]. This is also consistent with the observation that strong variants are cured more slowly by repression of Sis1 than weak variants [12]. [*RNQ*^+^] variants also exhibit differential sensitivity to Sis1 truncations and deletions. However, there are two differences from [*PSI*^+^]: 1) there are no clear correlations between the properties of specific variants maintained or eliminated and the particular constructs of Sis1 and 2) the requirement for the G/F region of Sis1 is absolute among examined [*RNQ*^+^] variants [65,66]. Several illuminating ideas have arisen from these studies. One is that prion variant requirements for Sis1 can be fully separated and mutually exclusive in some cases [40,66], even when tested simultaneously within the same cells [40]. This observation revealed that differences in prion maintenance observed among prion variants cannot simply be explained by positing differential sensitivities of prion variants for a general Sis1 function, which may be accomplished to different degrees by some truncations vs. others. Rather, this observation necessitates that Sis1 must have at least two (and possibly more) biochemically distinct functions that some prion variants require and others do not. These functions were localized to the glycine-rich regions of Sis1 and may be alternative modes of binding [40]. Another particularly illuminating finding regarding Sis1 function was the elucidation of multiple Sis1 binding sites on the Rnq1 protein, which forms [*RNQ*^+^] [24]. These sites differ in importance in the propagation of distinct variants with numerous examples of single-site or double-site mutations maintaining and/or eliminating different combinations of variants, often in a manner that was also sensitive to Sis1 expression level, supporting the notion that the mutations reduce Sis1 affinity specifically [24].

#### 5.1.2. Sis1 Orthologs in Other Species

Sis1 orthologs from several other species have also been examined. All rescue the lethality of a ∆*sis1* strain and allow the propagation of strong but not weak variants of [*PSI*^+^]; these include the human ortholog Hdj1/DNAJB1 [40,66], the *Drosophila melanogaster* ortholog Droj1 [67], and the *Arabidopsis thaliana* ortholog atDjB1 [68]. One study also found variant-specific impacts of the human ortholog Hdj1 on the propagation of [*RNQ*^+^] [66]. Another study examined the effects of disease-causing, G/F-region mutations of the human protein DNAJB6. When these mutations were made in the G/F region of Sis1, they again found variant-specific effects on variants of both [*PSI*^+^] and [*RNQ*^+^] [65]. Finally, another study found that overexpression of DNAJB6 itself cured weak but not strong variants of [*PSI*^+^] [69]. 

#### 5.1.3. Examples of Yeast Genetic Background Effects, Variant Switching, and Changes in Variant Dominance

Some examples of variant-specific Sis1 interactions have also been tied to the genetic background of the yeast, indicating that results are influenced by an uncharacterized genetic polymorphism in a particular yeast strain background. For example, three different weak [*PSI*^+^] variants exhibited atypical curing kinetics in just one of two examined yeast genetic backgrounds in one study, yet no effect was observed with three strong [*PSI*^+^] variants [12]. A similar example of a specific interaction between a single genetic background and a single prion variant was observed in experiments attempting to replace Sis1 with Hdj1 to support Hsp104-mediated prion elimination [70], a phenomenon addressed in a later section. 

Finally, one study found that deletion of Sis1’s G/F region caused a permanent change in one [*RNQ*^+^] variant [64], akin to other observations of [*RNQ*^+^] variant switching as a result of chaperone alterations to the Hsp90 system [71], discussed in a later section. Similarly, another study found that overproduction of Sis1, or expansion of its G/M-rich region, could alter the dominance of one prion variant over another, again with variant-specific effects, and again only in certain yeast genetic backgrounds. Specifically, Sis1 overexpression caused [*PSI*^+^]^VL^ dominance over [*PSI*^+^]^VK^ only in the 74-D694 background [56]. Expanding the G/M section to include 8 G/M rich repeats resulted in [*PSI*^+^]^VL^ dominance in the same background, but zero, three, and four repeats did not affect dominance [56]. All of these observations underscore the importance of reproducing experiments with multiple yeast prion variants and in multiple genetic backgrounds whenever feasible.

### 5.2. Other Hsp40s: Ydj1, Apj1, and Swa2

Apart from Sis1, 12 other J-proteins are at least partially present in the yeast cytosol, where they may interact with prion aggregates directly [72]. Screens deleting each J-protein gene individually have demonstrated that only Sis1 is essential for propagation of strong variants of [*PSI*^+^], a stable but otherwise uncharacterized variant of [*RNQ*^+^], and weak variants of [*PSI*^+^] [4,67]. Despite this, three other J-proteins—Ydj1, Swa2, and Apj1—have all been implicated at one point or another in prion biology. One investigation found an essential role for Ydj1, the most abundant J-protein in the yeast cytosol, in the maintenance of the prion [*SWI*^+^], but only one [*SWI*^+^] variant has been investigated to date [39]. Variant-specific effects of Ydj1 overexpression on other prions have been observed. Bradley et al. found that overexpression of Ydj1 cured some variants of [*RNQ*^+^]/[*PIN*^+^] [22]. However, probably because the strength of the [*PIN*^+^] phenotype is not clearly correlated to stability in mitosis or fiber fragility (as already discussed), the impact of Ydj1 overexpression exhibited fairly cryptic behavior as “high” variants of [*PIN*^+^] exhibited the greatest degree of prion curing, “medium” [*PIN*^+^] variants showed less, and “very high” and “low” variants of [*PIN*^+^] exhibited no curing [22]. Ydj1 overexpression alone has not been reported to cure strong or weak variants of [*PSI*^+^], despite numerous investigations. The simultaneous co-overexpression of Ydj1 and Ssa1 did cause some curing of weak, but not strong, variants of [*PSI*^+^] in one report [59]. Although, as also noted above in a previous section, Ssa1 overexpression alone has been found to cause curing of weak variants [51,53]. Finally, one investigation found that truncated Ydj1 constructs with specific mutations in the glycine-rich regions (Ydj1-134^G70N^ and Ydj1-134^Y66H^) could replace Sis1 to support cell viability and strong, but not weak, [*PSI*^+^] maintenance [73], again underscoring that strong [*PSI*^+^] variants require very little from Sis1 for propagation relative to weak [*PSI*^+^] variants and other prions [4,40,66,67,74,75].

The same 12 J-protein deletion screen described above also revealed a role for Apj1 in the variant-specific curing of [*PSI*^+^] by overexpression of Hsp104 (discussed below in another section) [67]. This screen also found an essential role for Swa2 in the propagation of [*URE3*]; as with [*SWI*^+^], only one variant of [*URE3*] was investigated [76,77]. Apj1 was also identified in a screen for factors that cured a synthetic prion, garnering its name “Anti-prion DnaJ” [78]. Swa2 is the yeast homolog of mammalian auxilin, which is involved in the disassembly of clathrin lattices [79]. Swa2’s role in prion biology, however, was shown to be independent of its interaction with clathrin and likely involves Hsp90 [76,77]. Despite these observations clearly linking these J-proteins to prion propagation, no information is known about any variant-specific interactions for either Apj1 or Swa2 in prion maintenance.

## 6. Sse1 (Hsp110, Nucleotide Exchange Factor)

Sse1 is a member of the Hsp110 chaperone class, which acts as a nucleotide exchange factor for Ssa. Only three studies have examined the impact of prion variation on requirements for Sse1. Fan et al. found that deletion of *SSE1* increased the size of strong [*PSI*^+^] aggregates resolved by SDDAGE, whereas this gene deletion cured strains bearing a weak variant of [*PSI*^+^] [80]. This is in agreement with the findings of Kryndushkin and Wickner, who also found that deletion of *SSE1* eliminated weak, but not strong, [*PSI*^+^] variants [81]. One likely explanation for these results is that Sse1 enhances the efficiency of the Sis1/Ssa/Hsp104 triad in fragmenting [*PSI*^+^] aggregates and that either fragmentation of weak variants is impossible without Sse1 NEF activity, or it is simply reduced to the point at which it no longer keeps pace with cell division. Deletion or overexpression of Sse1 cures one variant of [*URE3*], though differential effects among [*URE3*] variants have yet to be observed [81]. Finally, Lancaster et al. found that deletion of *SSE1* altered some but not all variant-dependent phenotypes of certain [*RNQ*^+^] variants, although most of these changes reverted once *SSE1* was reintroduced, indicating that in most cases the variant itself was not permanently altered [71].

## 7. Hsp90 and its Cochaperones

Hsp82 and Hsc82 are the two members of the Hsp90 chaperone class in *S. cerevisiae*. Hsp90s are large, dimeric chaperones found ubiquitously in eukaryotic cells, and in nearly every intracellular compartment, that function in a wide array of cellular processes [82]. Hsp90 and/or its cochaperones are required for the efficient elimination of [*PSI*^+^] by overexpression of Hsp104, the curing of [*PSI*^+^] by Ssa1-21, and for the propagation of at least one variant of [*URE3*] [54,77,83,84]. To our knowledge, only one study to date has uncovered variant-related effects of alterations to Hsp90 and its cochaperones. In contrast to the reversible changes to [*RNQ*^+^] variant phenotypes noted above when *SSE1* was deleted, Lancaster et al. found permanent alterations of [*RNQ*^+^] variants occurred as a result of the deletion of the constitutively expressed Hsp90 gene *HSC82*, or multiple Hsp90 cochaperones (*CPR6*, *CPR7*, *SBA1*, and *AHA1*). Interestingly, these alterations indicated a complete switching from one variant to another as a result of the loss of function in Hsp90 chaperone system [71]. 

## 8. Hsp104-Mediated Prion Elimination

When Hsp104 is ectopically overexpressed, only the prion [*PSI*^+^] is efficiently eliminated in a highly debated mechanism (recently reviewed by three different groups: Cox and Tuite, Matveenko et al., and Greene et al. [85,86,87], but even more recent, see also: Astor et al. [67]). The role of prion variation in Hsp104-mediated elimination of [*PSI*^+^] has been investigated many times and generally indicates that weaker variants are easier to cure than stronger variants [10,55,67,88,89]. 

Derkatch et al. were the first to demonstrate that weak [*PSI*^+^] variants are cured more quickly than strong variants when Hsp104 is overexpressed [10]. This has been confirmed several times by other groups with different strong and weak variants [67,90]. Chernoff and colleagues investigated the loss of [*PSI*^+^] after mild heat shock (39 °C) and found that [*PSI*^+^] was destabilized for multiple generations even after returning the culture to optimal growth conditions (30 °C) [55]. In the investigation, it was noted that weaker variants were affected more potently than stronger variants [55].

The *Candida albicans* ortholog of Hsp104, when overexpressed, is capable of efficiently curing both strong and weak variants of [*PSI*^+^]. The weaker variants tested resulted in a greater loss of [*PSI*^+^] than the stronger variants after 10–12 generations post-Hsp104 induction [88]. In a separate study conducted 11 years later using different specific variants, both the *C. albicans* and the *Schizosaccharomyces pombe* orthologs of Hsp104 were found to cure cell populations of weak, but not strong, variants of [*PSI*^+^] [89]. Additionally, the same effects were observed using a modified Hsp104 construct where the NBD2 and the C-terminal domain are replaced with similar regions from the *E. coli* ortholog ClpB [89]. ClpB does not have a C-terminal acidic extension, thus demonstrating that the C-terminal acidic extension of Hsp104 is not required for the curing of weak variants of [*PSI*^+^] [89]. At the extreme end of the sensitivity of [*PSI*^+^] variants to Hsp104 activity are numerous variants that are only revealed when Hsp104’s ability to cure [*PSI*^+^] when overexpressed is specifically impaired by mutation [91]. These hidden [*PSI*^+^] variants are apparently cured by the basal level of Hsp104 anti-[*PSI*^+^] activity, indicating that the possibility of prion variation in the cell is bounded in several ways by chaperone activity, a notion discussed further below. 

Sis1 is also required at sufficient expression levels for Hsp104-mediated elimination of [*PSI*^+^] [92], and Sis1’s overexpression accelerates prion loss [93]. A recent investigation further explored the roles of Sis1 in this process and elucidated the involvement of two other J-proteins, revealing that the interactions of all three J-proteins are [*PSI*^+^] variant-specific [67]. A construct of Sis1 lacking the G/F region can support both strong and weak [*PSI*^+^] propagation but is deficient in substituting for Sis1 in Hsp104-curing of strong variants only. The low-abundance J-protein Apj1, which was recently implicated in the degradation of sumoylated proteins [94], is also required for Hsp104-mediated curing of strong variants of [*PSI*^+^] [67]. Overexpression of Ydj1 completely blocks the curing of strong variants of [*PSI*^+^]. Strikingly, however, neither deletion of *APJ1*, nor overexpression of Ydj1 (either alone or in combination) affects the curing of weak variants. These observations indicate that J-proteins may not be required at all for the curing of these variants and raise the possibility that the mechanism by which weak [*PSI*^+^] variants are cured may be significantly distinct, biochemically, from that of strong variants, a possibility which has not been significantly addressed in debates surrounding the mechanism by which Hsp104 cures [*PSI*^+^] [67].

## 9. Chaperone-Sorting Factors

Two yeast proteins, Btn2 and its paralog Cur1 (possibly members of the Hook family of proteins), have been implicated as general prion-curing factors. Overproduction of either protein destabilizes or cures distinct [*URE3*] variants [95,96], while normal levels cure variants that can arise only in their absence [96]. Notably, prion propagon numbers appear to be a critical aspect separating the variants most affected, indicating that these proteins may act by reducing prion propagon counts [95,96]. Cur1 can also cure an artificial prion and was found to affect Sis1 localization to the nucleus [97], revealing a potential mechanism by which Cur1 could reduce propagon numbers. This was further supported by correlations between curing and Sis1 relocalization and the ability to dose-dependently disrupt curing by simultaneously overproducing Sis1 [98]. There is, however, some debate as to whether the mechanism of prion elimination by Cur1 is due to relocalization of Sis1 (see Wickner et al., Wickner et al., and the recent review by Matveenko et al. for additional details [86,96,99]).

## 10. Amyloid Variation is Bounded by Chaperone Activities and Other Systems

It is overwhelmingly clear that prion-chaperone interactions are dramatically impacted by amyloid polymorphism. The variations in propagons per cell and sensitivities to ectopic chaperone expression found among prion variants may result from structural differences that restrict fragmentation. For example, less mitotically-stable variants may require greater chaperone intervention for fragmentation to keep pace with cell division, perhaps due to fewer salient chaperone binding sites for the Hsp40/70/110 system or other structural differences that may potentially reduce Hsp104 processivity. Indeed, among prions and prion variants there seems to be a strong correlation between low propagon numbers and relatively large intracellular aggregates, indicating that some prions/variants are more difficult to productively fragment [100].

**Figure 2:**
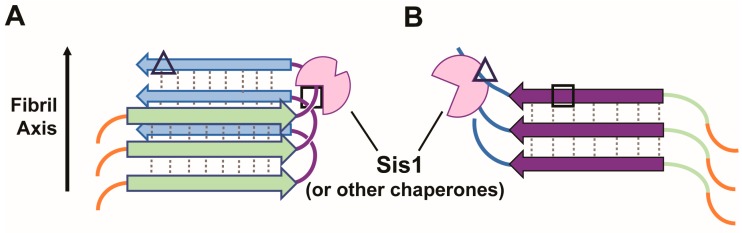
Prion variants may expose or obscure binding sites for distinct chaperone domains. Examples of two prion variants (“**A**” and “**B**”) in which different regions form the amyloid core, assumed here to be in-register parallel beta-sheet. The structural basis of yeast prion amyloids is debated, as is the structural nature of variants. See Wickner et al. for a recent review [99]. The solid arrow represents the direction of growth of the amyloid fibril, while dashed lines represent hydrogen bonds between parallel, in-register β-strands (horizontal arrows) that restrict the binding of chaperones to sites within the amyloidogenic regions. These sites (triangle and square) vary in structure when exposed and therefore may be recognized by distinct binding modes or domains of Sis1 and/or other chaperone proteins (pink cartoon). Thus, binding occurs only when the site is exposed, i.e., not part of the amyloid core.

Distinct amyloid conformers also likely limit the exposure of chaperone binding sites and/or alter the affinity of chaperones for exposed sites (due to changes in smaller scale structure). For example, mutually exclusive Sis1 requirements among prion variants have been observed [40,66], suggesting that Sis1 has more than one biochemical function or mode of binding that allows for the maintenance of distinct prion variants, which also differ in the binding sites that are competent for chaperone interaction [24]. The same may be true of interactions directly with Hsp104 [15]. These alternate binding sites may also interact with distinct chaperone domains (Figure 2). Variants are then lost from the cell population when the specific chaperone activity is disrupted. Furthermore, variants cannot re-form in a population if essential chaperone functions necessary for their stable propagation are not present. Thus, the chaperone functional diversity severely limits the number of possible amyloid structures that can form and propagate in a given cellular milieu (recently reviewed in the context of J-proteins in Killian and Hines [100]). These limits may be stretched by increasing and decreasing certain chaperone activities, resulting in new populations of variants that are able to propagate, as demonstrated by the discovery of new [*PSI*^+^] variants that are only observable when Hsp104 activity is either increased [41] or limited [91]. Indeed, additional cellular factors beyond just the chaperone complement also limit amyloid formation and propagation, termed anti-prion systems in yeast (See Wickner [101]).

Unweaving the complexity of the full “chaperome” interaction network is a Herculean task, and one that will likely never be complete, but work in this area has rapidly expanded. It is noteworthy that essentially everything now known about this network of relevant amyloid-chaperone interactions in yeast has been discovered in the past 25 years, most in just the last 10. Furthermore, the chaperone interactions of the majority of yeast prions remain completely unstudied, underscoring that despite our progress, there is still much left to be discovered.
